# Determinants of cyclization–decyclization kinetics of short DNA with sticky ends

**DOI:** 10.1093/nar/gkaa207

**Published:** 2020-04-13

**Authors:** Jiyoun Jeong, Harold D Kim

**Affiliations:** School of Physics, Georgia Institute of Technology, 837 State Street, Atlanta, GA 30332-0430, USA

## Abstract

Cyclization of DNA with sticky ends is commonly used to measure DNA bendability as a function of length and sequence, but how its kinetics depend on the rotational positioning of the sticky ends around the helical axis is less clear. Here, we measured cyclization (looping) and decyclization (unlooping) rates (*k*_loop_ and *k*_unloop_) of DNA with sticky ends over three helical periods (100-130 bp) using single-molecule fluorescence resonance energy transfer (FRET). *k*_loop_ showed a nontrivial undulation as a function of DNA length whereas *k*_unloop_ showed a clear oscillation with a period close to the helical turn of DNA (∼10.5 bp). The oscillation of *k*_unloop_ was almost completely suppressed in the presence of gaps around the sticky ends. We explain these findings by modeling double-helical DNA as a twisted wormlike chain with a finite width, intrinsic curvature, and stacking interaction between the end base pairs. We also discuss technical issues for converting the FRET-based cyclization/decyclization rates to an equilibrium quantity known as the J factor that is widely used to characterize DNA bending mechanics.

## INTRODUCTION

DNA under physiological conditions constantly undergoes conformational changes due to thermal fluctuations. Among those changes, bending motions coupled with twist can bring distal sites into proximity (1) and impact genome packaging and gene regulation (2,3). Some of these processes involve looped DNA segments much shorter than 500 bp, a length regime where the bending energy begins to dominate the free energy of loop formation. For example, some operons in *Escherichia coli*, such as *lac* and *gal*, are regulated by repressor proteins that form loops as small as ∼100 bp (4). Small DNA loops can also be induced by some restriction endonucleases (5–7) or actively extruded by chromosome packaging motor proteins (8). In these cases, proteins are thought to capture DNA loops that form spontaneously (4,9); therefore, it is of great importance to quantify the probability of spontaneous looping events. On the other hand, the protein complexes that bridge two distal sites of short DNA segments are subjected to a significant amount of bending and torsional stress depending on the loop geometry and size (10,11). This stress can affect the binding affinity of the protein complexes, and thereby alter the lifetime of the looped state (6,12,13). Recently, small DNA loops have also been used as force sensors and applicators to study bending mechanics of DNA itself or force-dependent conformational changes of other biomolecules (14–19). Therefore, measuring looping and unlooping dynamics of short DNA segments can give us insights into the energetics and internal forces that govern loop-associated processes and applications.

The simplest way to form DNA loops is to use DNA with two complementary single-stranded overhangs, or sticky ends, in a reaction called cyclization. In this reaction, the sticky ends of the same DNA molecule anneal to each other to form a ‘linker’ duplex. Under the assumption that the annealing reaction is independent of the mutual orientation between the sticky ends, the cyclization (looping) rate (*k*_loop_) is the product of just two quantities (20): (i) the effective concentration of one sticky end in the proximity of the other, which is known as the J factor (*J*), and (ii) the annealing rate constant between the two sticky ends (*k*_on_). Therefore, if *k*_on_ is known, the J factor can be determined by measuring *k*_loop_. The J factor can also be predicted from polymer models as a function of length, deformability, and loop geometry. Hence, the J factor has been used as a hallmark to test and refine DNA models such as the wormlike chain model (21,22).

Nonetheless, the experimental attempts to measure the J factor of DNA shorter than one persistence length (∼150 bp) have so far been controversial. Using a ligation-based assay, the Widom group first measured the J factor of short DNA molecules ∼100 bp in length (23). This study reported an anomalously high J factor, but the anomaly was soon proven by the Vologodskii group (24) to be an artifact due to the high concentration of ligase. In a more recent study, Vafabakhsh and Ha used a ligase-free fluorescence resonance energy transfer (FRET) assay to measure the J factor of short DNA molecules in the range between 50 and 200 bp (25). The reported J factor not only exceeded the wormlike chain model prediction, but also displayed a non-monotonic change as a function of DNA length. Considering FRET-based cyclization does not require torsional alignment between terminal base pairs, the origin of this non-monotonic length dependence remains unclear (26,27).

To shed light on this unresolved issue, we investigate how DNA cyclization and decyclization rates are influenced by the rotational positioning of the sticky ends around the helical axis and base stacking between the sticky ends. Using the single-molecule FRET assay introduced previously (28), we measured both cyclization (looping) and decyclization (unlooping) rates of short DNA (∼100 bp) over three helical periods. Our measurements show that the rotational positioning of sticky ends differentially affects cyclization and decyclization rates. We find that decyclization strongly depends on the stacking interaction between end base pairs, and as a result, the decyclization rate oscillates with a period of one helical turn, fast at half-integer number of helical turns and slow at integer number of helical turns. On the other hand, the cyclization rate is rather insensitive to such stacking interaction, which suggests that the main determinant of cyclization is the close proximity of the sticky ends. Nevertheless, the cyclization rate exhibits a peculiar length-dependent undulation, which could be due to the intrinsic curvature possessed by the DNA molecule. Based on our results, we present a three-state model for the FRET-based cyclization assay and discuss inherent uncertainties in the experimentally derived J factor that may hamper an accurate comparison to theory, especially for DNA shorter than 100 bp.

## MATERIALS AND METHODS

### Preparation of DNA molecules with sticky ends

Sticky-ended DNA with a FRET donor (Cy3), a FRET acceptor (Cy5), and a biotin was made in three steps. In Step 1, we obtained two sets of DNA molecules, DNA sets 1 and 2, by polymerase chain reactions (PCR) from two unrelated sources, phage lambda DNA and yeast genomic DNA, respectively (see Supplementary Table S1 for the list of DNA sequences in DNA sets 1 and 2). Annealing sites of PCR primers were carefully selected to generate molecules with different lengths, ranging from 96 to 134 bp for DNA set 1 and from 94 to 134 bp for DNA set 2 in steps of 2 bp. By PCR primer design, all PCR products shared common 20-bp ‘adaptor’ sequences at the ends.

**Figure 1.**
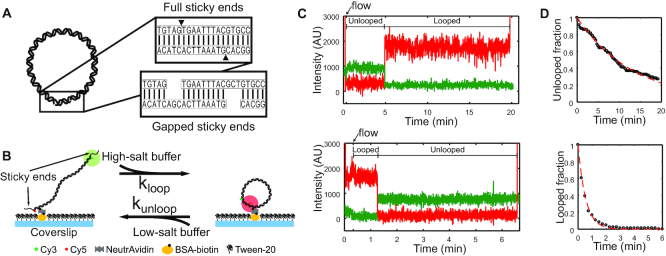
(**A**) Schematic representation of a looped DNA molecule with annealed sticky ends. Close-up views show duplexed sticky ends, which we refer to as a linker duplex, without (top) and with (bottom) gaps. (**B**) Experimental setup in the FRET-based cyclization (looping) and decyclization (unlooping) assays. Fluorescently labeled DNA molecules with sticky ends are immobilized on a passivated coverslip and continuously excited by the evanescent wave of a 532-nm laser. The cation concentration of the surrounding imaging buffer is exchanged to promote either looping or unlooping of the DNA molecules. (**C**) Examples of typical fluorescence trajectories of a single DNA molecule on the surface transitioning from the unlooped state to the looped state (top) and from the looped state to the unlooped state (bottom) upon sudden salt-exchange at time = 20 s (marked by an arrow). The green and red lines represent the donor (Cy3) and acceptor (Cy5) intensities, respectively. The molecules are briefly excited by a 640-nm laser in the beginning and the end for co-localization of Cy3 and Cy5 as well as to confirm the presence of Cy5. (**D**) Examples of decay curves of the unlooped (top) and looped (bottom) fractions of molecules. The rates are extracted by fitting the data (black) with an exponential function (red).

In Step 2, we performed two separate PCR reactions using the products from Step 1 as templates, with primers carrying the adaptor sequences and the necessary modifications (i.e. FRET donor, FRET acceptor, and biotin, see Supplementary Table S1 for details) (29). One set of PCR reactions produced donor-labeled DNA molecules with the sticky-end extension at the donor end. Another set of PCR reactions produced acceptor-labeled DNA molecules with the sticky-end extension at the other end. For gapped sticky ends, a stretch of three noncomplementary bases were inserted in the extensions (Figure 1A). The donor and the acceptor molecules were linked to the thymine bases nearest to the 5′-ends so that sticky-end annealing generated a high FRET signal (∼0.8).

Finally, in Step 3, strand exchange was performed between the donor-labeled and acceptor-labeled DNA from Step 2 by incubating the mixture (∼100 nM of Cy3-labeled DNA and ∼25 nM of Cy5-labeled DNA) at 95°C for 5 min and gradually cooling to the room temperature. As a result of strand exchange, the majority (∼70%) of products contained all the necessary modifications as well as the 5′ protruding sticky ends. A schematic summarizing this three-step protocol can be found in Supplementary Figure S1.

All of the PCR primers were commercially synthesized by Eurofins MWG Operon and Integrated DNA Technology (IDT) to at least HPLC-purity grade to minimize truncation or deletion errors. We used Mfold (30) to ensure that each sticky end does not form unintended secondary structures. We also note that all of the PCR products in each step were inspected by gel electrophoresis and extracted from the gel using a gel extraction/clean-up kit.

### FRET cyclization/decyclization assay

Here, we briefly summarize the flow-cell preparation protocol used in this study. A microscope slide with pre-drilled holes and a coverslip were cleaned by sonication in deionized water. After sonication, the slide and the coverslip were completely dried in a vacuum chamber for 15 min and etched in a plasma cleaner for additional 5 min. A dust-free, smooth surface was obtained at this stage. Then, the slide and the coverslip were silanized in a dichlorodimethylsilane (DDS)-hexane solution as previously described (31). After silanization, the flow-cell was assembled by joining the slide and the coverslip using double-sided tape and epoxy glue. The flow-cell was passivated and functionalized by biotinylated BSA and Tween-20 before DNA molecules were injected for immobilization.

For cyclization experiments (25), we first incubated the molecules in an imaging buffer containing no NaCl for 10 min. We then started recording the FRET time trajectories of the molecules and perfused 30 μL of 1 M [NaCl] imaging buffer into the flow channel to induce looping (Figure 1B). Perfusion was controlled by a motorized syringe pump at a constant flow rate. The decyclization experiment was performed in the same manner except that the salt concentration in the imaging buffer was changed from 2 M [NaCl] to either 75 mM or 1 M [NaCl]. All imaging buffers contained the PCD–PCA oxygen scavenging system (32). Figure 1C shows typical fluorescence intensity trajectories of Cy3 and Cy5 from these experiments. The temperature of the flow channel was maintained at 20°C via an objective lens temperature controller at all times. Single-molecule fluorescence data were acquired on an objective-based TIR microscope with an EMCCD camera (DU-897ECS0-# BV, Andor) at a rate of 500 ms per frame for looping experiments and 100 ms/frame for unlooping experiments.

### Association and dissociation rates of the linker duplex

To measure the association rate (*k*_on_) between the sticky ends and the lifetime (τ_on_) of the linker duplex, we prepared four different partial DNA duplexes that are sticky on one end and blunt on the other (see Supplementary Table S1). Two of them contained full sticky ends, and the other two gapped sticky ends. Each sticky end was labeled with either Cy3 or Cy5. These partial duplexes were constructed by heating a mixture of complementary oligonucleotides to 95°C for 5 min and gradually cooling to 4°C. The final concentrations of the oligonucleotides were ∼10 μM. The products from this reaction were purified by native polyacrylamide gel electrophoresis (12%, 19:1 ratio of acrylamide to bisacrylamide in 1× TBE buffer) and extracted by ‘crush and soak’ followed by ethanol precipitation. The concentration of the purified product was estimated from the absorbance of the fluorescent label at its maximum absorbance wavelength. To measure *k*_on_, one of the partial duplexes was immobilized on the surface, the other partial duplex carrying the complementary sticky end was injected into the flow cell at a known concentration, and the appearance of FRET events was monitored. To measure τ_on_, linker duplexes were formed on the surface, dissociation was induced by salt exchange, and the disappearance of FRET was monitored.

### Data analysis

We used Matlab to extract time trajectories of FRET values from the immobilized molecules. The FRET efficiency, or signal, was calculated from the background subtracted intensities of the donor (*I*_D_) and acceptor molecules (*I*_A_) using *I*_A_/(*I*_A_ + *I*_D_). The FRET time trajectories were filtered by applying a two-point moving average and were fed to a Hidden Markov Model estimator (33) to determine the transition points between the ideal FRET levels. The first passage time to FRET transition (low-to-high for looping or association and high-to-low for unlooping or dissociation) was collected from each FRET trajectory. About 150–250 trajectories were used to build the decay curve (Figure 1D) and to extract rates (see Supplementary Method andSupplementary Figures S2 and S3).

### Computation of minimum energy conformation and estimation of looping rate

We used the rigid base pair model to calculate the deformation energy of DNA. A triad of axes are assigned to each rectangular base pair, two along the long and short axes of the rectangle, and one perpendicular to the rectangle. In this representation, the backbone strands of DNA run through the two corners of the longer side of the rectangle. The DNA trajectory is determined by applying tilt, roll and twist angle rotations (*x*_*i*_) to triad axes separated by the rise distance between base pairs. The deformation energy (*E*_bend_) of DNA with *N* base pairs is given by

(1) $$\begin{equation*} E_{bend}/k_{\rm B} T = \sum _{i=1}^{3N-1}\alpha _i(x_i-x_{0,i})^2 , \end{equation*}$$

where α_*i*_ is the stiffness parameter associated with base pair step angle *x*_*i*_, *x*_0, *i*_ is the corresponding ground-state deformation angle, and *k*_B_*T* is the thermal energy. We assume that a loop can be formed when the two sticky ends are brought within some capture distance, and the rate of loop formation has an Arrhenius dependence on the energy of this teardrop conformation. To compute the energy of this conformation, we minimized *E*_bend_ as a function of *x*_*i*_ using the gradient descent method in the presence of a stiff harmonic potential that constrains the distance between the two ends of the antiparallel backbone strands. For quick convergence to the planar teardrop conformation, a circular arc was used as the initial conformation. By varying the bending direction Θ_*i*_ of the initial circular arc trajectory, we are able to compute minimum energy conformations in different bending directions *E*_bend_(Θ_*i*_). Based on the transition state theory, we estimate the looping rate by

(2) $$\begin{equation*} k_\textrm {loop} \propto \sum _{\Theta _i}\exp \left(-\frac{E_{bend}(\Theta _i)}{k_{{\rm B}}T} \right). \end{equation*}$$

## RESULTS

Using the single-molecule FRET assay, we measured the cyclization rate (*k*_loop_) and decyclization rate (*k*_unloop_) of DNA near 100 bp in length. Cyclization or decyclization was triggered by a sudden increase or decrease in NaCl concentration. The FRET signals of single molecules were continuously monitored from the beginning moment of buffer exchange, and the first transition times in the FRET signals were collected to obtain mean lifetimes or rates.

### The looping rate increases nonmonotonically as a function of DNA length

**Figure 2.**
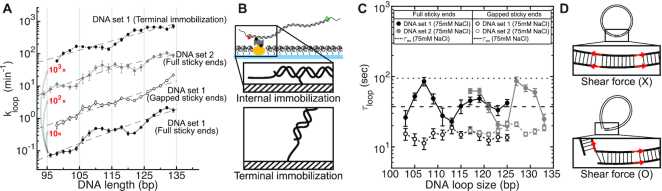
(**A**) Looping rates (*k*_loop_) of DNA molecules in DNA sets 1 and 2. For easy visualization, different data sets are vertically shifted by factors indicated in red. The closed and open circles represent data measured with the full and gapped sticky pairs, respectively. The dotted line represents data from DNA set 1 immobilized through the terminal phosphate. The dashed lines are quadratic fits to the data. Error bars represent the standard errors of the mean. (**B**) Different surface immobilization schemes. For most experiments, the DNA molecules were immobilized through a biotin linked to an internal thymine base (internal immobilization, top panel). This immobilization scheme can potentially hinder axial rotation of DNA, thus limiting the range of bending directions. In comparison, the terminal immobilization scheme (bottom panel) where DNA is attached to the surface through a terminal phosphate allows all bending directions to be explored for loop formation. (**C**) Mean lifetimes of DNA molecules in DNA sets 1 and 2 in the looped state. The DNA molecules with the full and gapped sticky ends are measured at 75 mM [NaCl] and shown as closed and open circles, respectively. The dotted and dashed horizontal lines represent the lifetimes of the full and gapped linker duplex, respectively. The DNA loop size includes the length of the annealed sticky ends (9 bp). Error bars represent the standard errors of the mean. (**D**) Schematic of how nick closing (terminal base stacking) can alter the stress geometry of the linker duplex. A fully stacked linker duplex (top) does not experience a shear force and therefore is more stable. In comparison, an unstacked linear duplex (bottom) experiences a shear force and therefore is less stable.

In Figure 2A, we present *k*_loop_ of molecules in DNA sets 1 and 2 whose lengths increase from 96 to 134 bp in 2-bp steps (excluding the sticky ends). This range spans more than three helical periods of DNA. Although DNA sets 1 and 2 contain completely unrelated sequences, both sets of DNA share a similar global length dependence with a >10-fold difference in *k*_loop_ between their shortest and the longest molecules. In all cases, *k*_loop_ tends to become slower with decreasing length, indicative of the increasing energy cost of looping.

Also plotted in Figure 2A is *k*_loop_ of DNA set 1 with gapped sticky ends, which prevent stacking between opposing terminal bases (34). DNA molecules with gapped sticky ends exhibit a similar dependence of *k*_loop_ on length. These molecules cyclize at a slightly slower rate due to the slower annealing rate of gapped sticky ends (see Supplementary Figure S4). These results indicate that stacking between the terminal bases of opposing sticky ends is not necessary to reach the looped state; that is, the transition state for the looped state is torsionally relaxed.

Interestingly, the length-dependent increase in *k*_loop_ is nonmonotonic and in some cases shows a clear undulation. For example, the bottom curve in Figure 2A shows steeper slopes at ∼106 and ∼126 bp. This undulation is seen in both DNA sets and with both internal and terminal immobilization schemes, and is more pronounced with full sticky ends. This undulatory pattern is quite different from the ∼10-bp oscillatory pattern observed in the ligation-based cyclization profile due to DNA torsion (21,35). This pattern vanishes in the presence of a 3-bp central mismatch (see Supplementary Figure S5), which suggests that it requires a coherent helical-phase difference between the positions of the sticky ends.

### The role of base stacking in the stability of DNA loop

Next, we present the dependence of decyclization kinetics on DNA length and sticky-end type. In Figure 2C, we plot the lifetime of the looped state, τ_loop_, which is the inverse of the decyclization rate (*k*_unloop_). Compared to cyclization kinetics, decyclization kinetics of DNA with full sticky ends exhibit a clear length-dependent oscillatory pattern with a period close to one helical turn of DNA (indicated by solid symbols). The oscillation is seen with two unrelated DNA sequences (black and gray symbols) and in two different salt conditions, 75 mM and 1 M [NaCl] (see Supplementary Figure S6 for data at 1 M). In both salt conditions, the period of oscillation is similar to one helical period of DNA (∼10.5 bp). At 1 M [NaCl], local maxima are identified at ∼105 and ∼115 for DNA set 1, and ∼127 for DNA set 2 (Supplementary Figure S6). These values are closer to integer multiples of the helical period (105, 115.5, 126 bp) than half-integer multiples. The exact positions of the peaks can reveal the helical period of a bent duplex, which is predicted to be longer than the unperturbed helical repeat due to twist-bend coupling (36,37). At 75 mM [NaCl], the locations of maxima (and minima) shift toward slightly larger values, which reflects an increase in the helical period. This change is consistent with unwinding of DNA (38,39) or larger twist-bend coupling due to weaker ionic screening (36,40). In addition to the oscillation phase, salt influences τ_loop_ and its oscillation amplitude (Supplementary Figure S6).

On the other hand, DNA loops captured with gapped sticky ends do not show length-dependent oscillation in τ_loop_ (open circles in Figure 2C). Moreover, τ_loop_ with gapped sticky ends was found to be similar in magnitude to the local minima of τ_loop_ with full sticky ends. Since the difference between full sticky ends and gapped sticky ends is the ability of base stacking (34,41), we reasoned that the oscillation seen with full sticky ends arises primarily from the stacking–unstacking equilibrium at the nicks in the loop; integer loops (loops with integer number of helical turns) are longer-lived than half-integer loops because of more stable base stacking. The salt dependent changes of τ_loop_ and the oscillation amplitude are also consistent with stabilization of base stacking at the nicks (42). In comparison, the lifetimes (τ_on_) of an unstressed linker duplex produced from bimolecular association of full and gapped sticky ends are plotted in Figure 2C as dotted are dashed lines, respectively. The local maxima and minima of τ_loop_ (filled circles) are consistently lower than the dotted and dashed lines, respectively, which reflects the fact that a shear stress accelerates melting of short DNA duplexes (Figure 2D) (43–45).

## DISCUSSION

In this study, we measured *k*_loop_ and *k*_unloop_ of DNA molecules over a length range of three helical periods using the FRET cyclization assay. We found that *k*_loop_ and *k*_unloop_ have two distinct length-dependent profiles. Most notably, *k*_unloop_ showed a clear oscillation with a period of one helical turn. The oscillation vanished in the absence of stackable terminal bases, thus revealing the role of the stacking–unstacking equilibrium at the nicks. On the other hand, *k*_loop_ showed a peculiar length-dependence where a slight change in slope was visible roughly every 20 bp. Such undulation was observed at least from two unrelated DNA sequences and with different surface immobilization schemes. In this section, we provide our explanations for these experimental findings and discuss our work in relation to previous DNA cyclization studies.

### Undulation of *k*_loop_

**Figure 3.**
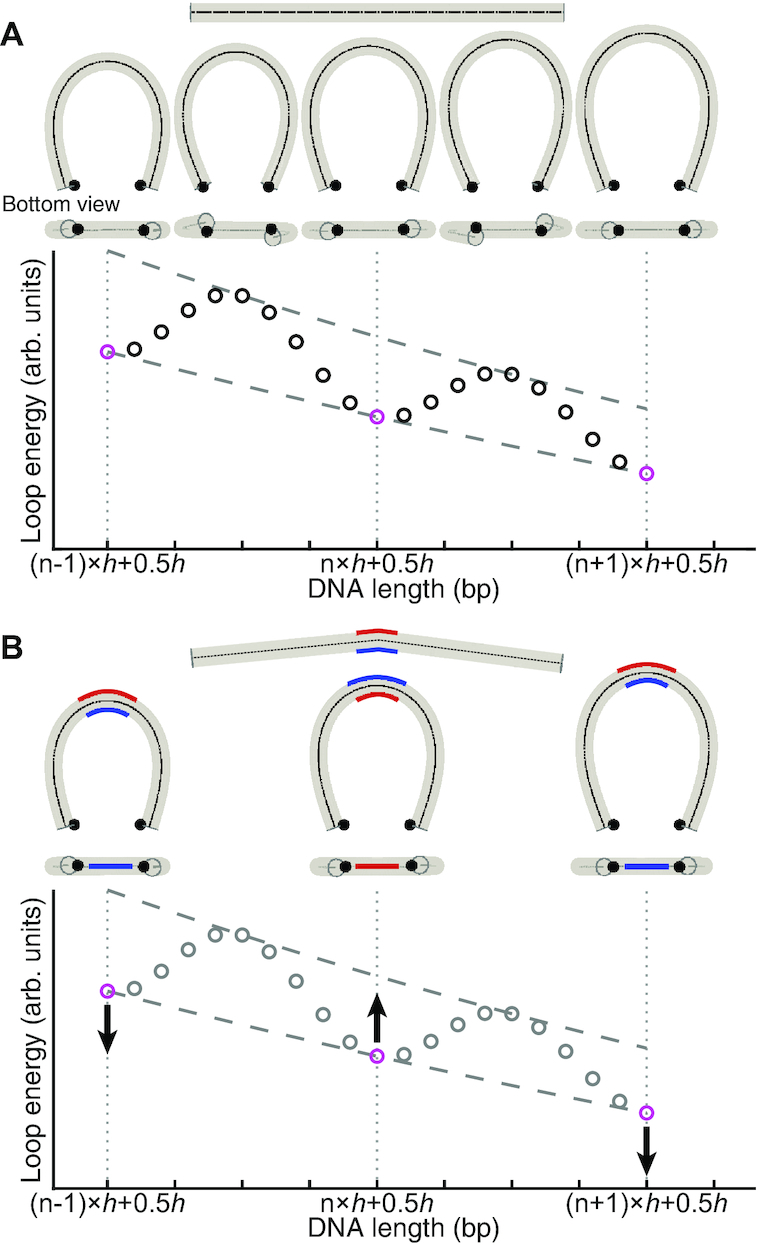
Illustration of the length-dependence of looping rate (*k*_loop_). (**A**) Looping of a straight DNA molecule. Shown below the straight DNA molecule (top) are the minimum energy loop conformations at integer (*n*-1,*n*, *n*+1) and half-integer (*n*-0.5,*n*+0.5,*n*+1.5) multiples of the helical turn (*h*). In this minimization, the constraint is imposed on the distance between the two sticky end positions (black dots). As shown in the bottom plot, the loop energy exhibits local minima (magenta circles) at half-integer multiples of h because the sticky ends can be on the near sides of a planar loop. At integer multiples of *h*, however, the minimum energy conformation is nonplanar, and the loop energy exhibits local maxima. (**B**) Looping of a curved DNA molecule. For illustrative purposes, nonzero intrinsic curvature is added to the center of the molecule (top), and the three conformations at half-integer multiples of *h* from (**A**) are shown. Due to the intrinsic curvature, the loop energy is lowered when the sticky ends are on the same side as the blue side, but increases when they are on the opposite (red) side. Hence, the intrinsic curvature can modulate the loop energy as a function of length with a period of 2*h*.

If DNA is modeled as a one-dimensional wormlike chain, the minimum energy conformation of the looped state has a teardrop shape, and the energy of this conformation will monotonically decrease with increasing chain length. Based on the transition state theory, *k*_loop_ is thus expected to monotonically increase with DNA length. To introduce undulation to *k*_loop_ versus length, we consider the finite width of the DNA helix and rotational positions of the sticky ends. As shown in Figure 3A, for DNA with a half-integer number of helical turns, the minimum energy conformation is planar with the sticky ends facing inward with respect to the bulk of DNA. For DNA with an integer number of helical turns, however, the minimum energy conformation of this bulky DNA is nonplanar under the same distance constraint on the sticky ends. Constrained minimization of this three-dimensional DNA model thus introduces a ∼10-bp oscillation to *k*_loop_ versus length with local maxima at half-integer helical turns and minima at integer helical turns (bottom plot, Figure 3A). Such oscillation is observed in a recent computational study (46). To explain the 20-bp periodicity observed in this study, we consider a more realistic DNA model which possesses nonzero curvature in the relaxed state (Figure 3B). With this intrinsic curvature, the energy of the looped state depends not only on how much DNA is bent, but also on which direction it is bent; the energy of loop formation would be lower if DNA bends ‘in phase’ with the intrinsic curvature, but would be higher if DNA bends in the opposite direction. As the DNA length is increased symmetrically at both ends, the bending direction of the curved region in reference to the loop changes, making a full turn every two helical turns. Therefore, the loop energy would be modulated at the period of two helical turns. This two-helical-turn (2*h*) oscillation due to intrinsic curvature interferes with the one-helical-turn (1*h*) oscillation due to the rotational positioning of the sticky ends. Based on our heuristic model, the amplitude of 2*h* oscillation is expected to be comparable to that of the 1*h* oscillation when the intrinsic curvature of the DNA molecule amounts to deflection of its axis by one helix-width from end to end.

To test this idea, we quantitatively explored the relationship between *k*_loop_ and length using a sequence-dependent base pair step model (47) that introduces nonzero intrinsic curvature. We first calculated the minimum energy (*E*_bend_) of the looped state as a function of bending direction or the rotational register angle (Θ) (48) of the first base pair step (Supplementary Figures S7 and S8). Based on the transition state theory, we estimate *k*_loop_ at length *L* according to

(3) $$\begin{equation*} k_\textrm {loop}(L) \propto \sum _{0<\Theta <2 \pi }\exp \left(-\frac{E_{bend}(\Theta ,L)}{k_{{\rm B}}T} \right) \textrm {.} \end{equation*}$$

*E*_bend_(Θ, *L*) calculated from zero-curvature and nonzero-curvature models are compared in Supplementary Figure S8. Interestingly, nonzero curvature can give rise to a ∼20-bp oscillation in length-dependence of *E*_bend_, which results in ∼20-bp periodicity of *k*_loop_ (Supplementary Figure S9).

In the review process, a question was raised as to whether surface-immobilization of DNA through in an internal base can somehow cause the 20-bp oscillation of *k*_loop_ by constraining the axial rotation of DNA. To address this question, we designed DNA molecules that are immobilized through the terminal phosphate group so that DNA can rotate around its axis and bend in any rotational register angle. Despite these additional degrees of freedom, we observed a similar 20-bp oscillation in *k*_loop_ (dotted line in Figure 2A), which suggests that 20-bp oscillation does not arise from surface confinement per se. Interestingly, the new DNA attachment scheme resulted in a noticeable phase shift in *k*_loop_ versus length. We speculate that this phase shift is due to the restriction in the rotational register angle: DNA cannot bend into the surface. Consistent with this speculation, if we sum over only a half interval of register angles to mimic the effect of surface hindrance, the resulting *k*_loop_ is phase-shifted (Supplementary Figure S9).

### Oscillation of *k*_unloop_

**Figure 4.**
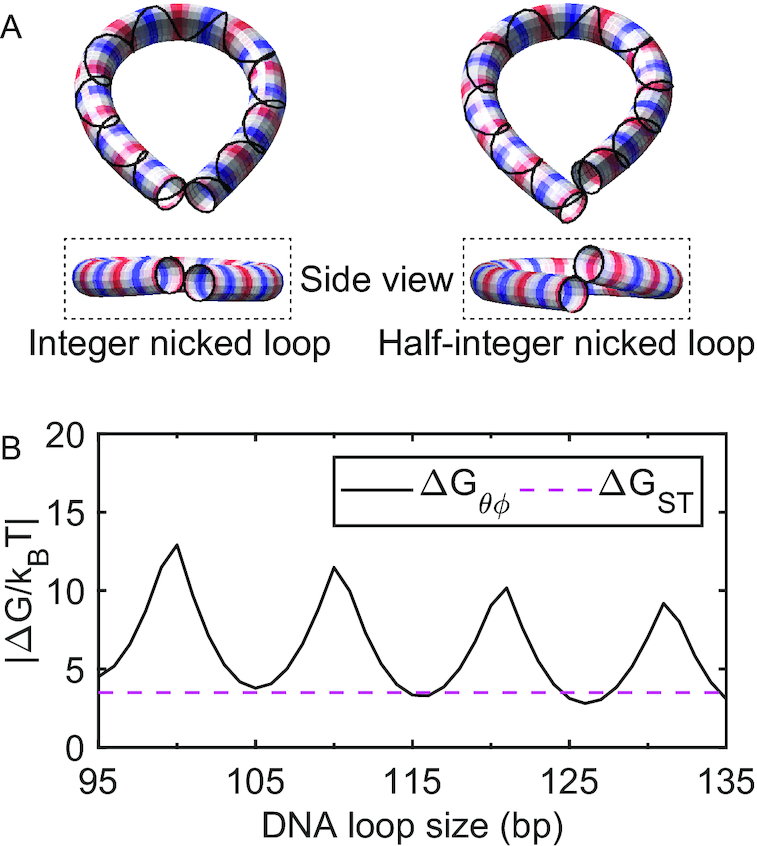
(**A**) Minimum-energy shapes of a cylindrical DNA model with a single nick (left: 105 bp and right: 100 bp). The strand without a nick is represented as a solid line around the cylindrical shapes. Here, the two ends (the first and last 10 bp) of the cylinder are volume-excluded from each other during the energy minimization procedure. (49). The alternating red and blue colors indicate one helical turn (e.g. the spacing between neighboring reds (or blue) is about one helical turn). (**B**) Comparison of free energy costs. Δ*G*_θϕ_ is the free energy cost to axially and torsionally align the ends of the helix at the tip of a small teardrop loop (solid line), and Δ*G*_ST_ is the average base pair stacking energy of all 16 dinucleotides taken from Ref. (50) (dashed line). The extrapolation method in the same reference is applied to extrapolate the stacking energy for 20°C and [NaCl] = 0.1 M.

To gain insight into the shorter lifetimes of half-integer loops than integer loops, we compute the minimum energy conformations of integer and half-integer loops with a single open nick (49). For this calculation, we used the same cylindrical DNA model as in Figure 3 and applied a zero-distance constraint to two ends of the same DNA strand. As shown in Figure 4A, integer loops adopt a nearly planar teardrop shape, whereas half-integer loops are nonplanar. Therefore, nick closing which requires axial and torsional alignment at the apex of the teardrop would be energetically more challenging to half-integer loops. We can estimate the free energy cost (Δ*G*_θϕ_) for the teardrop loop to achieve axial (θ) and torsional (ϕ) alignment at the nick from the J factors according to

(4) $$\begin{equation*} \Delta G_{\theta \phi } = - k_{\rm B} T \log (J_{\theta \phi }/J) , \end{equation*}$$

where *J*_θϕ_ and *J* are the semi-analytically derived J factors with and without the helical alignment, respectively (51,52). As predicted, Δ*G*_θϕ_ of half-integer loops is much larger than that of integer loops (solid line, Figure 4B). Δ*G*_θϕ_ of integer loops is still much larger than the thermal energy, but is comparable to the free energy (Δ*G*_ST_) of base stacking (dashed line, Figure 4B) which we estimated from the literature (50). In agreement with our thermodynamic argument, a recent coarse-grained simulation (46) also shows that half-integer loops adopt a non-planar teardrop loop configuration in which base stacking across nicks is disrupted.

### Kinetic scheme for FRET-based cyclization

**Figure 5.**
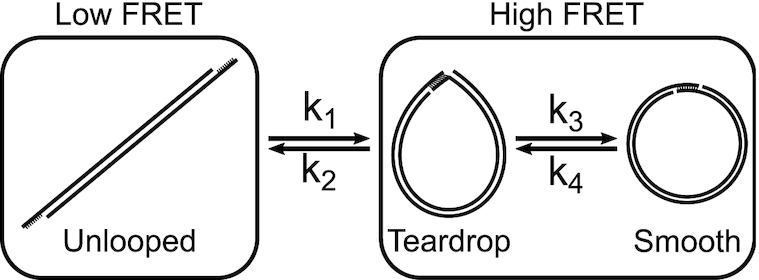
Three-state DNA cyclization model. A sticky-ended short DNA molecule undergoes a transition between the low FRET (unlooped) state and the high FRET (looped) state. The transition rates between these two FRET states (*k*_1_ and *k*_2_) are governed by the bending energy of DNA. Two different macrostates, teardrop and smooth, can exist within the high FRET state since the looped molecule contains nicks that can spontaneously close and open. Transitions between the teardrop and smooth states occur with rates of *k*_3_ and *k*_4_, and are associated with local transitions of nick closing and opening. For the transition from the teardrop state to the smooth state, integer loops need axial alignment only while half-integer loops need both axial and torsional alignment. Therefore, integer loops can transition to the smooth state more readily than half-integer loops.

Although indistinguishable by FRET, our kinetic analysis distinguishes two primary macrostates in the looped state and thus validates a three-state cyclization model (Figure 5): (i) unlooped, (ii) teardrop loop (end juxtaposed) and (iii) smooth loop (axially and torsionally aligned, and terminally stacked). A looped state with two open nicks is also possible, but is omitted from the model because it is significantly less favorable than the other two looped states (see Supplementary Results and Discussion). The looped state is first captured in a torsionally relaxed state (*k*_loop_ = *k*_1_), which is likely a teardrop loop with an open nick(s). Thus, transitions (*k*_1_, *k*_2_) between the first two states involve bending and base pairing between the sticky ends. In contrast, transitions (*k*_3_, *k*_4_) between the second and third states depend on bending, twist, and stacking energies. This equilibrium explains the difference in τ_loop_ between integer and half-integer loops. Integer loops only require in-plane bending fluctuations to close the nick, while half-integer loops require energetically demanding out-of-plane deformations to do so. Therefore, half-integer loops would be stalled in the teardrop state most of the time, and decyclize at a rate of *k*_unloop_ = *k*_2_, while integer loops would be rapidly equilibrated between teardrop and smooth states, and decyclize at a slower rate of $k_\textrm {unloop}\cong k_\textrm {2}\cdot \frac{k_\textrm {4}}{k_\textrm {3}+k_\textrm {4}}$ (see Supplementary Results and Discussion for derivation.). Based on this insight, we propose the oscillation amplitude and phase in *k*_unloop_ versus DNA length (Figure 2C) as a useful measure to probe twist-bend coupling and torsional stiffness of DNA in different sequence contexts or experimental conditions.

This three-state model also reveals the key difference of ligation-free cyclization (53,54) from ligation-based cyclization (24,35). The ligation-free cyclization measures *k*_1_, the transition rate to the composite of teardrop and smooth states, and is proportional to *J*. In comparison, the ligation-based cyclization measures the rate of intramolecular ligation, which is proportional to the equilibrium fraction of the smooth state (21) or *J*_θϕ_. *J*_θϕ_ changes by a few orders of magnitude over one helical period due to the strong torsional dependence of *k*_3_ and *k*_4_, whereas *J* or *k*_1_ is predicted to be free of such torsional modulation.

### Revisiting the J factor of short DNA

The wormlike chain model is widely successful in describing the statistical mechanics of long DNA. However, whether it correctly describes the looping probability of DNA shorter than 100 bp is still debated. The comparison between measurement and model is most comprehensively shown on the plot of the J factor versus DNA length, called the cyclization profile (55). The experimental J factor (*J*_exp_) is extracted from dynamic measurements under the assumption of fast looping/unlooping equilibrium followed by a slow capture step such as ligation or annealing. In comparison, the theoretical J factor (*J*_th_) is derived using equilibrium statistical mechanics. Using the single-molecule FRET assay, Vafabakhsh and Ha (25) obtained *J*_exp_ that becomes increasingly higher than *J*_th_ below 100 bp. In the same study (25), *J*_exp_ was shown to change nonmonotonically with length, but it is unclear whether this change is related to the oscillation seen in ligation-based cyclization studies (24,26,27).

**Figure 6.**
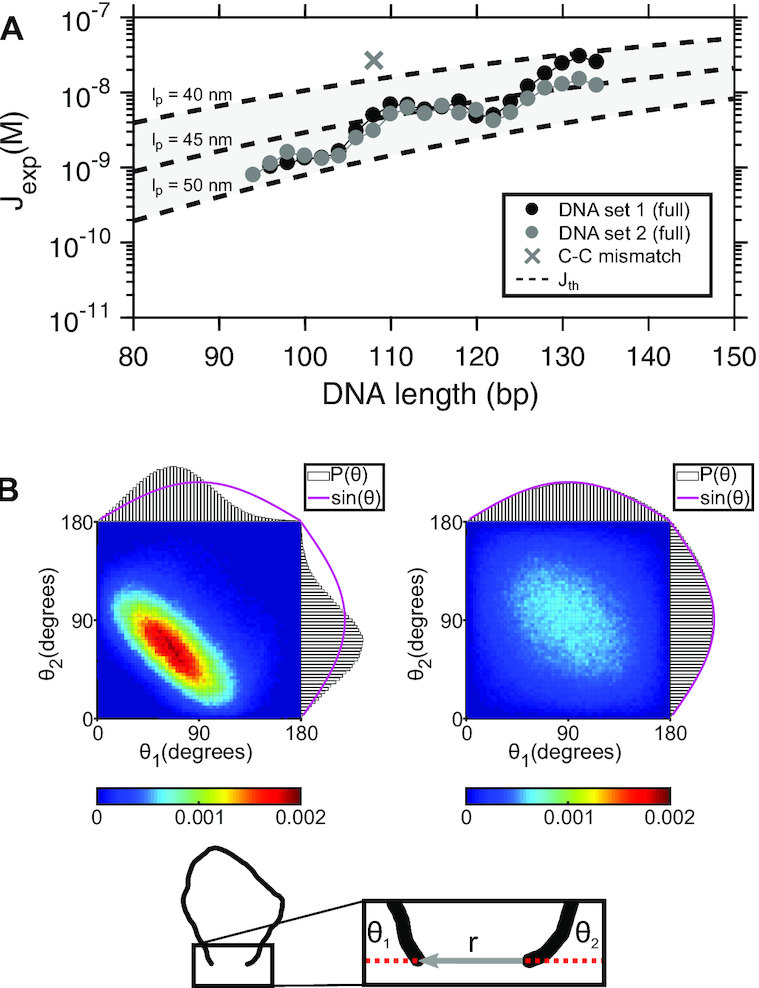
(**A**) *J*_exp_ as a function of DNA length. The measured values of *J*_exp_ from DNA sets 1 and 2 with full sticky ends are plotted in black and gray circles, respectively. *J*_exp_ is compared with *J*_th_ (dashed lines) calculated based on (52). In this calculation, we assumed the loop capture distance is equal to 7 nm. The shared area between the dashed lines represent the prediction made with a range of persistence lengths from 40 to 50 nm. The size of error bars (not shown) is similar to the size of the data points. (**B**) Joint probability distributions (*P*(θ_1_, θ_2_)) of coarse-grained DNA chains. The schematic at the bottom shows a DNA chain constrained with a short end-to-end distance, |**r**|. θ_1_ and θ_2_ are the angles between the chain ends and the end-to-end vector. The left and right density plots represent the joint distributions of θ_1_ and θ_2_ for 100- and 500-bp loops, respectively. The projected probability distributions of θ_1_ and θ_2_ are individually plotted along the x- and y-axis of each density plot, respectively. The magenta line represents the unconstrained *P*(θ_1_, θ_2_), which is equal to the sine function.

Using *J*_exp_ = *k*_loop_/*k*_on_, we extracted *J*_exp_ of DNA sets 1 and 2 with full sticky ends using *k*_loop_ from Figure 2A and *k*_on_ measured from the bimolecular association of the full sticky ends (*k*_on_ = 1.196× 10^6^ M^−1^ s^−^^1^, see Supplementary Figure S4). The results are shown in Figure 6A (black and grey dots). In the same figure, we also plot *J*_th_ of a wormlike chain (52) using a range of persistence lengths from 40 to 50 nm and a capture distance of 7 nm (dashed lines). *J*_th_ plotted here should be taken as a lower limit as it relies on a loosely-defined capture distance (46) and approximates the fluctuations about the minimum-energy loop only up to the quadratic terms (56). We also note that a wormlike chain with nonzero curvature is predicted to have a higher *J*_th_ than an isotropic wormlike chain (57). Nevertheless, as shown in this plot, *J*_exp_ of DNA sets 1 and 2 correspond to persistence lengths between 40 and 49 nm. The variability in the values of persistence length is mainly due to the length-dependent undulation of *k*_loop_ explained earlier but is still within the accepted range of experimentally determined values (58). The difference in *J*_exp_ between the two DNA sets is not remarkable considering that *J*_exp_ can vary with sequence by a few orders of magnitude in the similar length range (59). In comparison, we show that a single base pair mismatch in the center of a 108-bp DNA can increase *J*_exp_ by almost 10-fold (cross (×) in Figure 6A). Based on the cyclization profile alone, it is difficult to conclude whether DNA looping is wormlike or not in this length range between 95 and 135 bp. A more detailed DNA model that includes helical geometry and intrinsic curvature may be necessary to quantitatively explain the measured cyclization profile.

### Limitations of the J factor below 100 bp

Although understanding energetics of DNA looping at even shorter lengths (<100 bp) is of growing interest, we argue that the J factor is neither a theoretically relevant nor an experimentally accessible quantity in this regime. As DNA becomes shorter, the end effects become more significant: end base pairs are more prone to fraying (46,60,61), and end segments are more flexible than internal segments (62). Moreover, discreteness of base pairs and sequence-dependent effects such as curvature (63) cannot be sufficiently averaged out over several helical turns. Therefore, the J factor which describes the average behavior of a continuous, homogeneous polymer is no longer relevant in this length scale. A recent simulation-based study clearly demonstrates the systematic deviation of the J factor of a more realistic DNA model from that of a one dimensional wormlike chain (46).

As DNA becomes shorter, the fraction of molecules that loop on a laboratory time scale becomes extremely small, and detection of this trace amount in a bulk ligation assay becomes quite laborious and cumbersome (24). In this aspect, the single-molecule FRET assay seems to be a better alternative to extract *J*_exp_. However, we argue that *J*_exp_ extracted from the FRET assay becomes a bad proxy for *J*_th_. If the second-order annealing rate constant (*f*) between the two sticky ends in cyclization is identical to that in bimolecular association (*k*_on_), one can use *k*_on_ to cancel out the annealing rate constant *f* in *k*_loop_ and recover the looping probability density *J*_th_:

(5) $$\begin{equation*} J_\textrm {exp}=\frac{k_\textrm {loop}}{k_\textrm {on}} = \frac{f}{k_\textrm {on}}J_\textrm {th}=J_\textrm {th} \textrm {.} \end{equation*}$$

However, if *f* depends on the relative orientation of the sticky ends, *f* is no longer equal to *k*_on_ because the relative orientation of the sticky ends is highly restrained for short DNA. To highlight this effect, we plot the joint probability distribution of two angles (*P*(θ_1_, θ_2_)) formed between the end-to-end vector and the helical axes of the end segments (Figure 6B) using a Monte Carlo simulation of a wormlike chain (64). These angles thus represent how much the two sticky ends would have to deviate from the helical axes for annealing. Large angles will incur some energetic penalty because dangling bases in the sticky ends can stack (65,66), albeit weakly. The two angles at which two separate molecules encounter would be independent and uniformly distributed, and therefore, (*P*(θ_1_, θ_2_)) should be proportional to sin θ_1_sin θ_2_ (magenta lines, Figure 6B). A similar distribution is obtained for the ends of a 500-bp DNA, significantly longer than the persistence length (right, Figure 6B). For the ends of short DNA (100 bp), however, the two angles are highly restrained because of the strong bending stress in the looped DNA (left, Figure 6B)). Hence, for 100-bp or shorter DNA, *f* = *k*_on_ is no longer valid, and *J*_exp_ deviates from *J*_th_.

The FRET cyclization assay of short DNA suffers from practical complications as well. Extremely slow events are inevitably masked by photobleaching of the fluorophores, which leads to an overestimation of *k*_loop_ and *J*_exp_. This overestimation becomes more severe as cyclization becomes slower, as much as 25% for the 94-bp DNA we tested in this study. In our experience, slower cyclization kinetics are also fitted more poorly with a single exponential function, possibly due to an increasing inactive fraction over time (see Supplementary Method). Therefore, *J*_exp_ of DNA shorter than 100-bp carries substantial experimental and statistical uncertainties. In our opinion, *J*_exp_ of short DNA should be interpreted only as a comparative measure of loopability, but not as a proxy for *J*_th_.

## CONCLUSION

The single-molecule FRET assay (25,54) can detect cyclization intermediates without the need of protein mediated ligation and is thus thought to be a more accurate method to measure the intrinsic looping probability of short DNA. However, exact boundary interactions and loop geometry of these intermediates are not known, which complicates the interpretation of the apparent cyclization rate. In this study, we measured cyclization and decyclization rates of short DNA as a function of DNA length. Cyclization and decyclization rates exhibit a clear difference in length-dependence. We present a three-state cyclization model that is kinetically and thermodynamically consistent with our data and existing stacking free energy parameters, and propose the cyclization and decyclization profiles as new measures to explore intrinsic curvature and twist-bend coupling of DNA. Lastly, the J factors extracted from cyclization rates of 94–134-bp DNA correspond to persistence lengths in the range of 40–49 nm. However, given that the J factor extracted by the FRET assay exhibits an unexpected nonmonotonic length dependence in some conditions, and the angle dependence of the sticky-end annealing rate is not known, the apparent discrepancy between the experimental and theoretical J factors should not be over-interpreted as an anomaly in DNA bending mechanics.

## Supplementary Material

gkaa207_Supplemental_File

## Notes

Present address: Jiyoun Jeong, Wyss Institute for Biologically Inspired Engineering at Harvard University, Boston, MA 02115, USA and Department of Systems Biology, Harvard Medical School, Boston, MA 02115, USA.

## References

[1] Tardin C. The mechanics of DNA loops bridged by proteins unveiled by single-molecule experiments. Biochimie. 2017; 142:80–92. 28804000 10.1016/j.biochi.2017.08.002

[2] Brennan L.D., Forties R.A., Patel S.S., Wang M.D. DNA looping mediates nucleosome transfer. Nat. Commun. 2016; 7:13337. 27808093 10.1038/ncomms13337 PMC5097161

[3] Becker N.A., Schwab T.L., Clark K.J., Maher L.J. III Bacterial gene control by DNA looping using engineered dimeric transcription activator like effector (TALE) proteins. Nucleic Acids Res. 2018; 46:2690–2696. 29390154 10.1093/nar/gky047 PMC5861415

[4] Cournac A., Plumbridge J. DNA looping in prokaryotes: experimental and theoretical approaches. J. Bacteriol. 2013; 195:1109–1119. 23292776 10.1128/JB.02038-12 PMC3591992

[5] Gemmen G.J. DNA looping by two-site restriction endonucleases: heterogeneous probability distributions for loop size and unbinding force. Nucleic Acids Res. 2006; 34:2864–2877. 16723432 10.1093/nar/gkl382 PMC1474071

[6] Laurens N., Rusling D.A., Pernstich C., Brouwer I., Halford S.E., Wuite G.J.L. DNA looping by FokI: the impact of twisting and bending rigidity on protein-induced looping dynamics. Nucleic Acids Res. 2012; 40:4988–4997. 22373924 10.1093/nar/gks184 PMC3367208

[7] Shang Y., Zhang N., Zhu P., Luo Y., Huang K., Tian W., Xu W. Restriction enzyme cutting site distribution regularity for DNA looping technology. Gene. 2014; 534:222–228. 24211387 10.1016/j.gene.2013.10.054

[8] Ganji M., Shaltiel I.A., Bisht S., Kim E., Kalichava A., Haering C.H., Dekker C. Real-time imaging of DNA loop extrusion by condensin. Science. 2018; 360:102–105. 29472443 10.1126/science.aar7831 PMC6329450

[9] Marko J.F., De Los Rios P., Barducci A., Gruber S. DNA-segment-capture model for loop extrusion by structural maintenance of chromosome (SMC) protein complexes. Nucleic Acids Res. 2019; 47:6956–6972. 31175837 10.1093/nar/gkz497 PMC6649773

[10] Zhang Y., McEwen A.E., Crothers D.M., Levene S.D. Statistical-mechanical theory of DNA looping. Biophys. J. 2006; 90:1903–1912. 16361335 10.1529/biophysj.105.070490 PMC1386771

[11] Mulligan P.J., Chen Y.-J., Phillips R., Spakowitz A.J. Interplay of protein binding interactions, DNA mechanics, and entropy in DNA looping kinetics. Biophys. J. 2015; 109:618–629. 26244743 10.1016/j.bpj.2015.06.054 PMC4572505

[12] Chen Y.-J., Johnson S., Mulligan P., Spakowitz A.J., Phillips R. Modulation of DNA loop lifetimes by the free energy of loop formation. Proc. Natl. Acad. Sci. U.S.A. 2014; 111:17396–17401. 25411314 10.1073/pnas.1415685111 PMC4267329

[13] Yan Y., Leng F., Finzi L., Dunlap D. Protein-mediated looping of DNA under tension requires supercoiling. Nucleic Acids Res. 2018; 46:2370–2379. 29365152 10.1093/nar/gky021 PMC5861448

[14] Shroff H., Sivak D., Siegel J.J., McEvoy A.L., Siu M., Spakowitz A., Geissler P.L., Liphardt J. Optical Measurement of Mechanical Forces Inside Short DNA Loops. Biophys. J. 2008; 94:2179–2186. 18065484 10.1529/biophysj.107.114413 PMC2257878

[15] Qu H., Wang Y., Tseng C.-Y., Zocchi G. Critical torque for kink formation in double-stranded DNA. Phys. Rev. X. 2011; 1:021008.

[16] Fields A.P., Meyer E.A., Cohen A.E. Euler buckling and nonlinear kinking of double-stranded DNA. Nucleic Acids Res. 2013; 41:9881–9890. 23956222 10.1093/nar/gkt739 PMC3834817

[17] Joseph C., Tseng C.-Y., Zocchi G., Tlusty T. Asymmetric effect of mechanical stress on the forward and reverse reaction catalyzed by an enzyme. PLoS One. 2014; 9:e101442. 25000118 10.1371/journal.pone.0101442 PMC4085160

[18] Mustafa G., Chuang C.-Y., Roy W.A., Farhath M.M., Pokhrel N., Ma Y., Nagasawa K., Antony E., Comstock M.J., Basu S. et al. A force sensor that converts fluorescence signal into force measurement utilizing short looped DNA. Biosens. Bioelectron. 2018; 121:34–40. 30195120 10.1016/j.bios.2018.08.073 PMC6151280

[19] Kostrz D., Wayment-Steele H.K., Wang J.L., Follenfant M., Pande V.S., Strick T.R., Gosse C. A modular DNA scaffold to study protein–protein interactions at single-molecule resolution. Nat. Nanotechnol. 2019; 14:988–993. 31548690 10.1038/s41565-019-0542-7

[20] Crothers D.M., Drak J., Kahn J.D., Levene S.D. DNA Bending, Flexibility, and Helical Repeat by Cyclization Kinetics. 1992; Elsevier. 10.1016/0076-6879(92)12003-9 1518450

[21] Peters J.P., Maher L.J. DNA curvature and flexibility in vitro and in vivo. Q. Rev. Biophys. 2010; 43:23–63. 20478077 10.1017/S0033583510000077 PMC4190679

[22] Levene S.D., Giovan S.M., Hanke A., Shoura M.J. The thermodynamics of DNA loop formation, from J to Z. Biochem. Soc. Trans. 2013; 41:513–518. 23514145 10.1042/BST20120324

[23] Cloutier T.E., Widom J. Spontaneous sharp bending of double-stranded DNA. Mol. Cell. 2004; 14:355–362. 15125838 10.1016/s1097-2765(04)00210-2

[24] Du Q., Smith C., Shiffeldrim N., Vologodskaia M., Vologodskii A. Cyclization of short DNA fragments and bending fluctuations of the double helix. Proc. Natl. Acad. Sci. U.S.A. 2005; 102:5397–5402. 15809441 10.1073/pnas.0500983102 PMC556251

[25] Vafabakhsh R., Ha T. Extreme bendability of DNA less than 100 base pairs long revealed by single-molecule cyclization. Science. 2012; 337:1097–1101. 22936778 10.1126/science.1224139 PMC3565842

[26] Vologodskii A., Du Q., Frank-Kamenetskii M.D. Bending of short DNA helices. Artif. DNA: PNA XNA. 2013; 4:1–3. 23406786 10.4161/adna.23892 PMC3654724

[27] Vologodskii A., Frank-Kamenetskii M.D. Strong bending of the DNA double helix. Nucleic Acids Res. 2013; 41:6785–6792. 23677618 10.1093/nar/gkt396 PMC3737528

[28] Le T.T., Kim H.D. Probing the elastic limit of DNA bending. Nucleic Acids Res. 2014; 42:10786–10794. 25122748 10.1093/nar/gku735 PMC4176374

[29] Le T.T., Kim H.D. Studying DNA looping by single-molecule FRET. J. Vis. Exp. 2014; 88:e51667. 10.3791/51667 PMC4208857 24998459

[30] Zuker M. Mfold web server for nucleic acid folding and hybridization prediction. Nucleic Acids Res. 2003; 31:3406–3415. 12824337 10.1093/nar/gkg595 PMC169194

[31] Hua B., Han K.Y., Zhou R., Kim H., Shi X., Abeysirigunawardena S.C., Jain A., Singh D., Aggarwal V., Woodson S.A. et al. An improved surface passivation method for single-molecule studies. Nat. Methods. 2014; 11:1233–1236. 25306544 10.1038/nmeth.3143 PMC4245390

[32] Aitken C.E., Marshall R.A., Puglisi J.D. An oxygen scavenging system for improvement of dye stability in single-molecule fluorescence experiments. Biophys. J. 2008; 94:1826–1835. 17921203 10.1529/biophysj.107.117689 PMC2242739

[33] Bronson J.E., Fei J., Hofman J.M., Gonzalez R.L., Wiggins C.H. Learning rates and states from biophysical time series: a Bayesian approach to model selection and single-molecule FRET data. Biophys. J. 2009; 97:3196–3205. 20006957 10.1016/j.bpj.2009.09.031 PMC2793368

[34] Kashida H., Kurihara A., Kawai H., Asanuma H. Orientation-dependent FRET system reveals differences in structures and flexibilities of nicked and gapped DNA duplexes. Nucleic Acids Res. 2017; 45:e105. 28369626 10.1093/nar/gkx200 PMC5499647

[35] Shore D., Baldwin R.L. Energetics of DNA twisting. J. Mol. Biol. 1983; 170:957–981. 6315955 10.1016/s0022-2836(83)80198-3

[36] Marko J.F., Siggia E.D. Bending and twisting elasticity of DNA. Macromolecules. 1994; 27:981–988.

[37] Haijun Z., Zhong-Can O.-Y. Bending and twisting elasticity: a revised Marko-Siggia model on DNA chirality. Phys. Rev. E. 1998; 58:4816.

[38] Taylor W.H., Hagerman P.J. Application of the method of phage T4 DNA ligase-catalyzed ring-closure to the study of DNA structure: II. NaCl-dependence of DNA flexibility and helical repeat. J. Mol. Biol. 1990; 212:363–376. 2319604 10.1016/0022-2836(90)90131-5

[39] Rybenkov V.V., Vologodskii A.V., Cozzarelli N.R. The effect of ionic conditions on DNA helical repeat, effective diameter and free energy of supercoiling. Nucleic Acids Res. 1997; 25:1412–1418. 9060437 10.1093/nar/25.7.1412 PMC146597

[40] Olsen K., Bohr J. Geometry of the toroidal N-helix: optimal-packing and zero-twist. New. J. Phys. 2012; 14:023063.

[41] Lane M.J., Paner T., Kashin I., Faldasz B.D., Li B., Gallo F.J., Benight A.S. The thermodynamic advantage of DNA oligonucleotide ‘stacking hybridization’ reactions: energetics of a DNA nick. Nucleic Acids Res. 1997; 25:611–616. 9016603 10.1093/nar/25.3.611 PMC146477

[42] Yakovchuk P., Protozanova E., Frank-Kamenetskii M.D. Base-stacking and base-pairing contributions into thermal stability of the DNA double helix. Nucleic Acids Res. 2006; 34:564–574. 16449200 10.1093/nar/gkj454 PMC1360284

[43] de Gennes P.-G. Maximum pull out force on DNA hybrids. Comp. Rend. ’Acad. Sci. - Ser. IV - Phys. 2001; 2:1505–1508.

[44] Whitley K.D., Comstock M.J., Chemla Y.R. Elasticity of the transition state for oligonucleotide hybridization. Nucleic Acids Res. 2016; 45:547–555. 27903889 10.1093/nar/gkw1173 PMC5314771

[45] Whitley K.D., Comstock M.J., Chemla Y.R. Ultrashort nucleic acid duplexes exhibit long wormlike chain behavior with force-dependent edge effects. Phys. Rev. Lett. 2018; 120:068102. 29481284 10.1103/PhysRevLett.120.068102

[46] Harrison R.M., Romano F., Ouldridge T.E., Louis A.A., Doye J.P. Identifying physical causes of apparent enhanced cyclization of short DNA molecules with a coarse-grained model. J. Chem. Theory. Comput. 2019; 15:4660–4672. 31282669 10.1021/acs.jctc.9b00112 PMC6694408

[47] Zuiddam M., Everaers R., Schiessel H. Physics behind the mechanical nucleosome positioning code. Phys. Rev. E. 2017; 96:052412. 29347769 10.1103/PhysRevE.96.052412

[48] Sanghani S.R., Zakrzewska K., Harvey S.C., Lavery R. Molecular modelling of (A4T4NN) n and (T4A4NN) n: sequence elements responsible for curvature. Nucleic Acids Res. 1996; 24:1632–1637. 8649979 10.1093/nar/24.9.1632 PMC145856

[49] Zhang Y., Crothers D.M. Statistical mechanics of sequence-dependent circular DNA and its application for DNA cyclization. Biophys. J. 2003; 84:136–153. 12524271 10.1016/S0006-3495(03)74838-3 PMC1302599

[50] Krueger A., Protozanova E., Frank-Kamenetskii M.D. Sequence-dependent basepair opening in DNA double helix. Biophys. J. 2006; 90:3091–3099. 16500982 10.1529/biophysj.105.078774 PMC1432109

[51] Shimada J., Yamakawa H. Ring-closure probabilities for twisted wormlike chains. Application to DNA. Macromolecules. 1984; 17:689–698.

[52] Douarche N., Cocco S. Protein-mediated DNA loops: effects of protein bridge size and kinks. Phys. Rev. E. 2005; 72:061902. 10.1103/PhysRevE.72.061902 16485969

[53] Wang J.C., Davidson N. On the probability of ring closure of lambda DNA. J. Mol. Biol. 1966; 19:469–482. 5969076 10.1016/s0022-2836(66)80017-7

[54] Le T.T., Kim H.D. Measuring shape-dependent looping probability of DNA. Biophys. J. 2013; 104:2068–2076. 23663850 10.1016/j.bpj.2013.03.029 PMC3647197

[55] Tong Y., Manning R.S. Quantifying the impact of simple DNA parameters on the cyclization J-factor for single-basepair-addition families. Sci. Rep. 2018; 8:doi:10.1038/s41598-018-22502-7. 10.1038/s41598-018-22502-7 PMC5861124 29559729

[56] Giovan S.M., Hanke A., Levene S.D. DNA cyclization and looping in the wormlike limit: normal modes and the validity of the harmonic approximation. Biopolymers. 2015; 103:528–538. 26014845 10.1002/bip.22683 PMC6815668

[57] Czapla L., Swigon D., Olson W.K. Sequence-dependent effects in the cyclization of short DNA. J. Chem. Theory. Comput. 2006; 2:685–695. 26626674 10.1021/ct060025+

[58] Brunet A., Tardin C., Salome L., Rousseau P., Destainville N., Manghi M. Dependence of DNA persistence length on ionic strength of solutions with monovalent and divalent salts: a joint theory–experiment study. Macromolecules. 2015; 48:3641–3652.

[59] Rosanio G., Widom J., Uhlenbeck O.C. In vitro selection of DNAs with an increased propensity to form small circles. Biopolymers. 2015; 103:303–320. 25620396 10.1002/bip.22608

[60] Cong P., Dai L., Chen H., van der Maarel J.R., Doyle P.S., Yan J. Revisiting the anomalous bending elasticity of sharply bent DNA. Biophys. J. 2015; 109:2338–2351. 26636945 10.1016/j.bpj.2015.10.016 PMC4675846

[61] Joffroy B., Uca Y.O., Prešern D., Doye J. P.K., Schmidt T.L. Rolling circle amplification shows a sinusoidal template length-dependent amplification bias. Nucleic Acids Res. 2017; 46:538–545. 10.1093/nar/gkx1238 PMC5778537 29237070

[62] Wu Y.-Y., Bao L., Zhang X., Tan Z.-J. Flexibility of short DNA helices with finite-length effect: from base pairs to tens of base pairs. J. Chem. Phys. 2015; 142:125103. 25833610 10.1063/1.4915539

[63] Marin-Gonzalez A., Vilhena J., Moreno-Herrero F., Perez R. DNA crookedness regulates DNA mechanical properties at short length scales. Phys. Rev. Lett. 2019; 122:048102. 30768347 10.1103/PhysRevLett.122.048102

[64] Jeong J., Le T.T., Kim H.D. Single-molecule fluorescence studies on DNA looping. Methods. 2016; 105:34–43. 27064000 10.1016/j.ymeth.2016.04.005 PMC4967024

[65] Freier S.M., Alkema D., Sinclair A., Neilson T., Turner D.H. Contributions of dangling end stacking and terminal base-pair formation to the stabilities of XGGCCp, XCCGGp, XGGCCYp, and XCCGGYp helixes. Biochemistry. 1985; 24:4533–4539. 4063336 10.1021/bi00338a008

[66] Bommarito S., Peyret N., SantaLucia J. Jr Thermodynamic parameters for DNA sequences with dangling ends. Nucleic Acids Res. 2000; 28:1929–1934. 10756193 10.1093/nar/28.9.1929 PMC103285

