# Supplementary material for: Determinants of cyclization–decyclization kinetics of short DNA with sticky ends
Source: Nucleic Acids Res. 2020 Apr 13;48(9):5147–56. doi: 10.1093/nar/gkaa207 (PMC7229855; doi:10.1093/nar/gkaa207)
Supplement: gkaa207_Supplemental_File [file gkaa207_supplemental_file.pdf]

Determinants of cyclization-decyclization kinetics of short DNA with sticky ends

Jiyoun Jeong and Harold D. Kim [1]
School of Physics, Georgia Institute of Technology, 837 State Street, Atlanta, GA 30332-0430, USA

Supplementary Method: Extracting rates from the decay curves

The looping and unlooping rates (k_{loop} and k_{unloop}) were extracted from fitting an exponential function to the unlooped and looped state decay curves, respectively. We observed that the looped state population decayed to zero within our typical recording time (~ 6 min) and therefore used an exponential function of the form $N(t) = N_0 \exp(-k_{\text{unloop}}t)$ for fitting. We found fitting with a bi-exponential function was not particularly better than a single exponential function (Figure S2). Unlike the looped state, the unlooped state population did not decay to zero even after 20 minutes of observation (Figure S3), which was our typical recording time. A similar observation was made in our previous study [1]. Thus, we fitted the decay curve with an equation of the form $N(t) = (1-N_\infty)\exp(-k_{\text{loop}}t)+N_\infty$, where N_∞ was taken to be the average unlooped fraction of 110-bp or longer DNA molecules at the end of the 30-min observation period ($N_\infty = 0.29 \pm 0.013$). For consistency, we used the same value of N_∞ for molecules shorter than 110 bp. The mean lifetimes, τ_{unloop} and τ_{loop}, are the reciprocals of k_{loop} and k_{unloop}, respectively.

Supplementary Results and Discussion: Free energy difference between the singly kinked and doubly-kinked loops

We replaced $J_{\varphi\theta}$ in Equation (4) in the main text with the J factor estimated with an end-to-end distance of 3 nm (i.e. the contour length of the linker duplex) to calculate the free energy difference between the singly and doubly-kinked loops. Taking into account ΔG_{ST} ($\approx$ 3.5 k_BT), we find that the doubly-kinked loop is thermodynamically less favorable than the singly-kinked loop by ~2 k_BT, which corresponds to an equilibrium fraction of 0.14, in the range between 95 bp and 125 bp. Therefore, we focus our discussion on transitions between the teardrop state with a single open nick and the smooth state.

Supplementary Results and Discussion: Derivation of k_{unloop}

The master equations for the evolution of the teardrop (P_t) and smooth (P_s) state probabilities are

$$dP_t/dt = -(k_2 + k_3)P_t + k_4 P_s$$
$$dP_s/dt = k_3 P_t - k_4 P_s$$

The characteristic equation of the transition matrix is

$$\lambda^2 + (k_2 + k_3 + k_4)\lambda + k_2 k_4 = 0$$

from which the fast rate (k_f) and slow rate (k_s) emerge:

$$k_f = \frac{(k_2 + k_3 + k_4)}{2}\left[1 + \sqrt{1 - \frac{4k_2k_4}{(k_2 + k_3 + k_4)^2}}\right]$$

$$k_s = \frac{(k_2 + k_3 + k_4)}{2}\left[1 - \sqrt{1 - \frac{4k_2k_4}{(k_2 + k_3 + k_4)^2}}\right]$$

Applying the initial condition that the composite (high FRET) state is in equilibrium ($k_3P_t(0) = k_4P_s(0)$), the time-dependence of the composite state is given by a double exponential decay

[1] Corresponding author. Email: harold.kim@physics.gatech.edu
Present address: Jiyoun Jeong, Wyss Institute for Biologically Inspired Engineering at Harvard University, Boston, MA 02115, USA and Department of Systems Biology, Harvard Medical School, Boston, MA 02115, USA

$$P_t(t) + P_s(t) = \frac{(k_f - (k_3 + k_4))k_s}{(k_f - k_s)(k_3 + k_4)} e^{-k_f t} + \frac{((k_3 + k_4) - k_s)k_f}{(k_f - k_s)(k_3 + k_4)} e^{-k_s t}$$

Based on the apparent single-exponential decay, we demand that the amplitude of the fast decay component be much smaller than that of the slow decay component.

$$(k_f - (k_3 + k_4))k_s \ll ((k_3 + k_4) - k_s)k_f$$

After using $k_s + k_f = k_2 + k_3 + k_4$and $k_s k_f = k_2 k_4$, one obtains

$$k_2(k_3 + k_4) + (k_3 + k_4)(k_3 + k_4) \gg 2k_2k_4 \quad (1)$$

For half-integer loops, $k_3 \ll k_4$

$$k_2k_4 + {k_4}^2 \gg 2k_2k_4$$

For the inequality to hold

$$k_4 \gg k_2$$

For integer loops, we have $k_3 \sim k_4$ based on our thermodynamic argument that teardrop and smooth loops are equally stable. Therefore, the second term on LHS of (1) must be greater than the first term:

$$k_3 + k_4 \gg k_2$$

In both cases, the pre-equilibrium assumption is valid

$$k_3 + k_4 \gg k_2,$$

and the slow rate can be approximated as

$$k_s \approx \frac{(k_3 + k_4)}{2}\left[1 - \left(1 - \frac{2k_2k_4}{(k_3 + k_4)^2}\right)\right] = k_2 \cdot \frac{k_4}{k_3 + k_4}$$

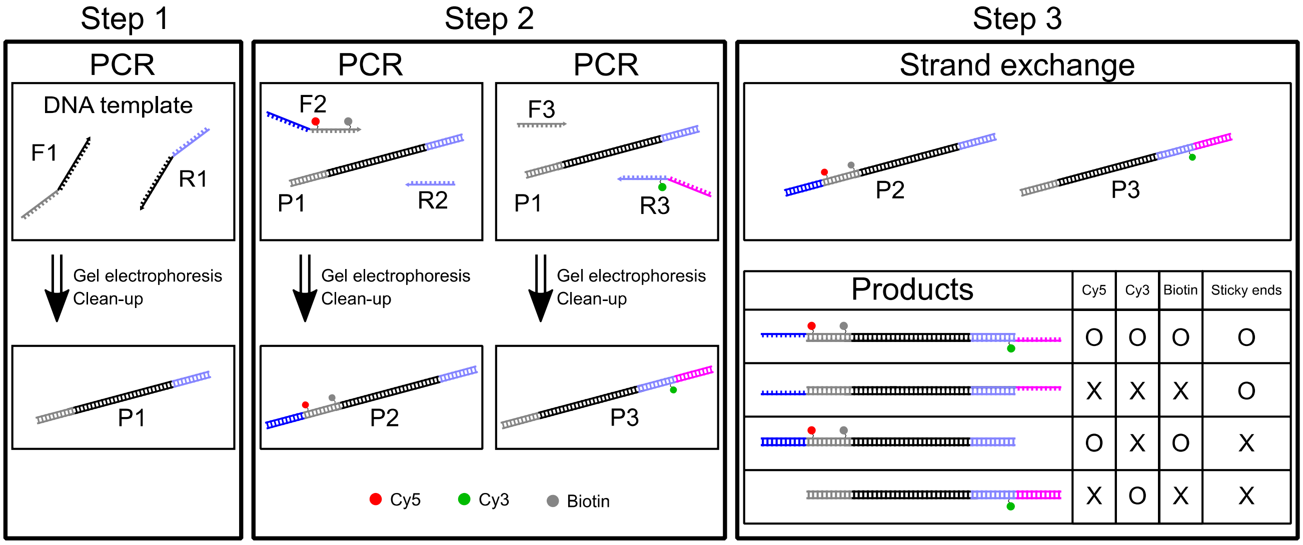

Supplementary Figure S1: Preparation of sticky-ended DNA with modifications (i.e. Cy3, Cy5, and biotin). In Step 1, short DNA is amplified from yeast genomic DNA or phage lambda DNA by PCR. The forward (F1) and reverse (R1) primers introduce the 20-bp adapter sequences (grey and purple). In Step 2, two separate PCR reactions are performed on P1 using two different PCR primer pairs (F2 + R2 and F3 + R3). The reaction with F2 and R2 adds the sticky-end extension (blue) at the Cy5-labeled side. The reaction with F3 and R3 adds the complementary sticky-end extension (magenta) at the Cy3-labeled side. In Step 3, the products from Step 2 (P2 and P3) are subjected to a strand exchange reaction, which results in the desired sticky-ended DNA plus three different by-products. All possible products from the strand exchange reaction in Step 3 are shown. The correct product is identified by colocalization of immobilized Cy3 and Cy5 spots.

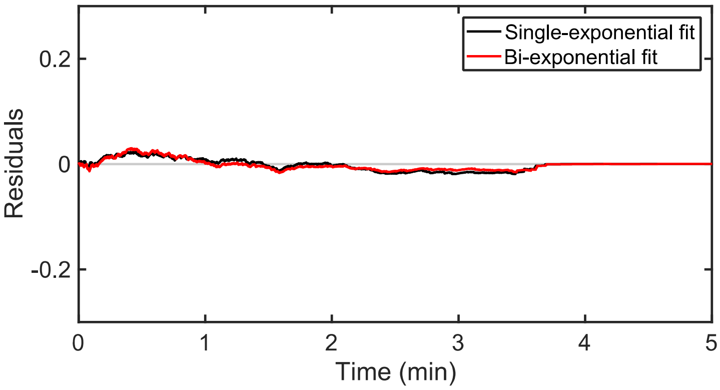

Supplementary Figure S2: Mean residuals of all DNA molecules from Figure 2(C) in the main text.

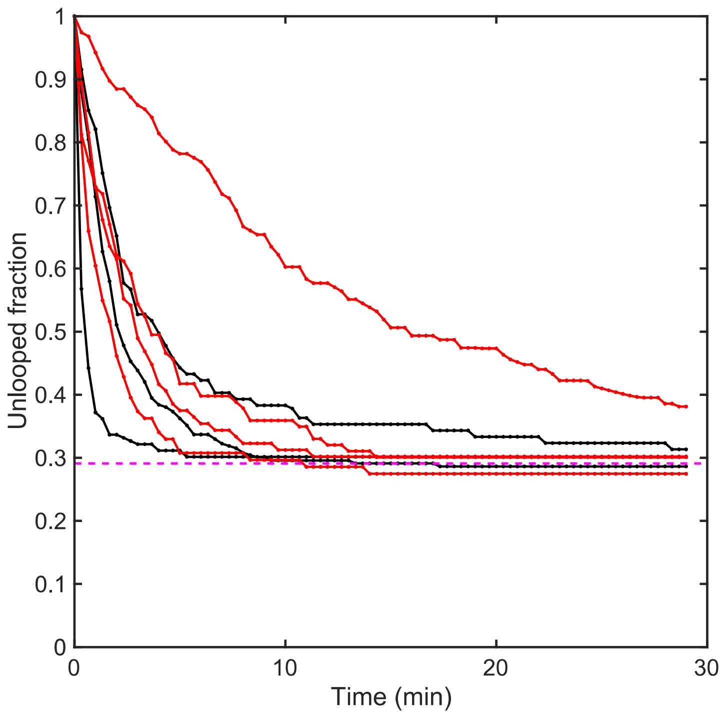

Supplementary Figure S3: Decay of the unlooped fraction. The black curves represent molecules from DNA set 1 with length equal to 108, 124, and 132 bp from top to bottom. The red curves represent DNA set 2 with length equal to 100, 110, 120, and 130 bp from top to bottom. The dashed horizontal line indicates the mean unlooped fraction (N_∞ = 0.29±0.013) determined from the molecules longer than or equal to 110 bp.

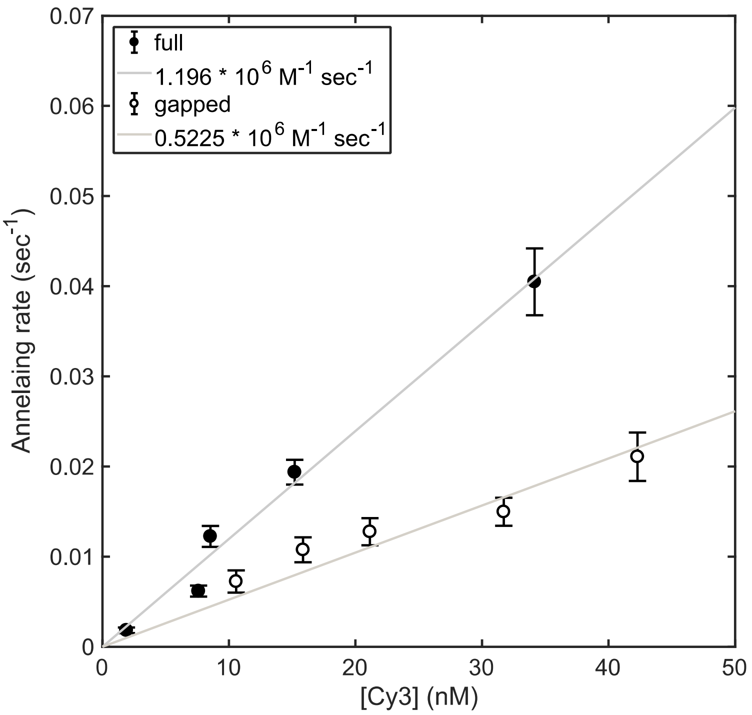

Supplementary Figure S4: Extracting k_{on}. The annealing rates of full and gapped sticky ends were measured at different concentrations of the Cy3-labeled molecules. k_{on} is found from the slope of the rate vs. concentration curve.

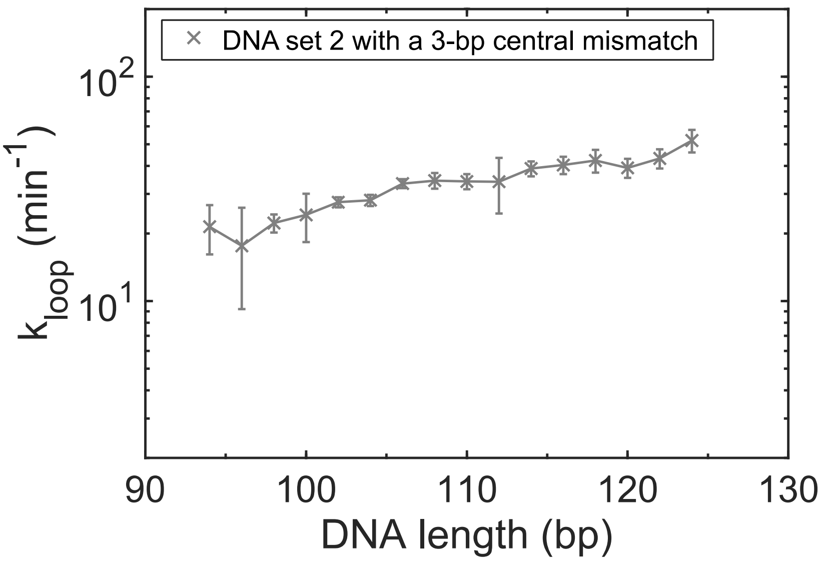

Supplementary Figure S5: k_{loop} of DNA set 2 in the presence of a 3-bp central mismatch. Error bars represent the standard errors of the mean.

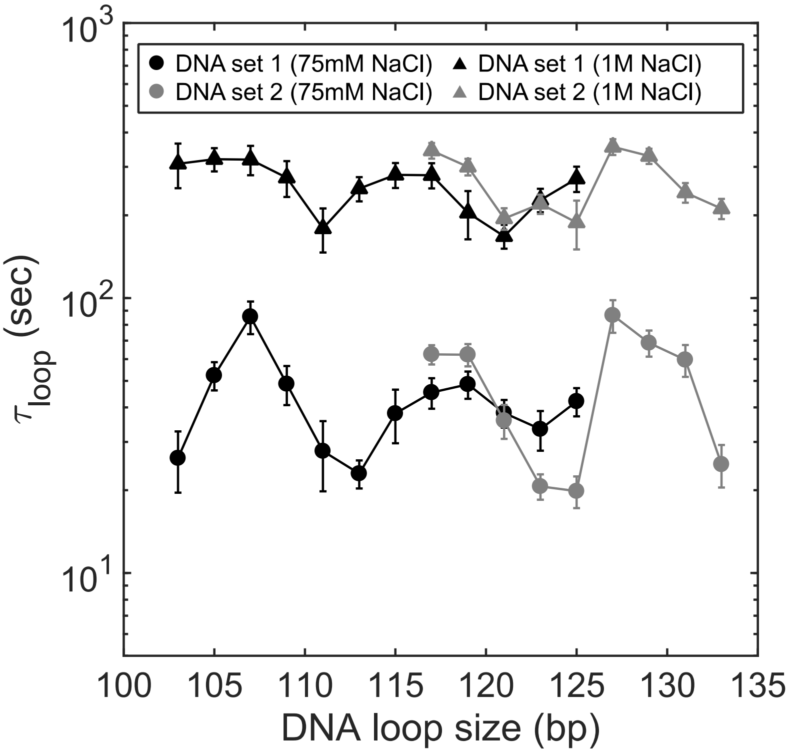

Supplementary Figure S6: Mean looped state lifetimes of DNA molecules in two different salt conditions, 75 mM and 1 M [NaCl]. Error bars represent the standard errors of the mean.

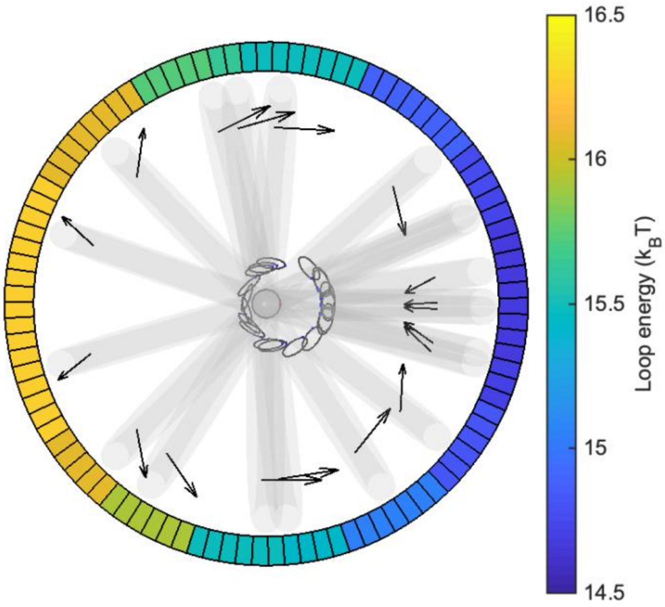

Supplementary Figure S7: Loop energy as a function of bending direction or the rotational register angle of the first base pair step. The first base pair is fixed on the xy-plane (the plane of the page), and different rotational register angles are sampled by varying the relative magnitudes of roll and tilt angles of the first base pair step. The minimum energy conformations in different rotational register angles are illustrated as gray cylinders. The top and bottom surfaces of the cylinder corresponding to the first and last base pairs are outlined. The red and blue dots represent the positions of the two sticky ends. The black arrows indicate the orientation of the central base pair step with respect to the first base pair step. The corresponding loop energies are presented as a circular heat map. The distance constraint is applied to the red and blue dots whereas the energy is calculated from the trajectory of the cylinder axis. As a result, the loop energy varies with rotational register.

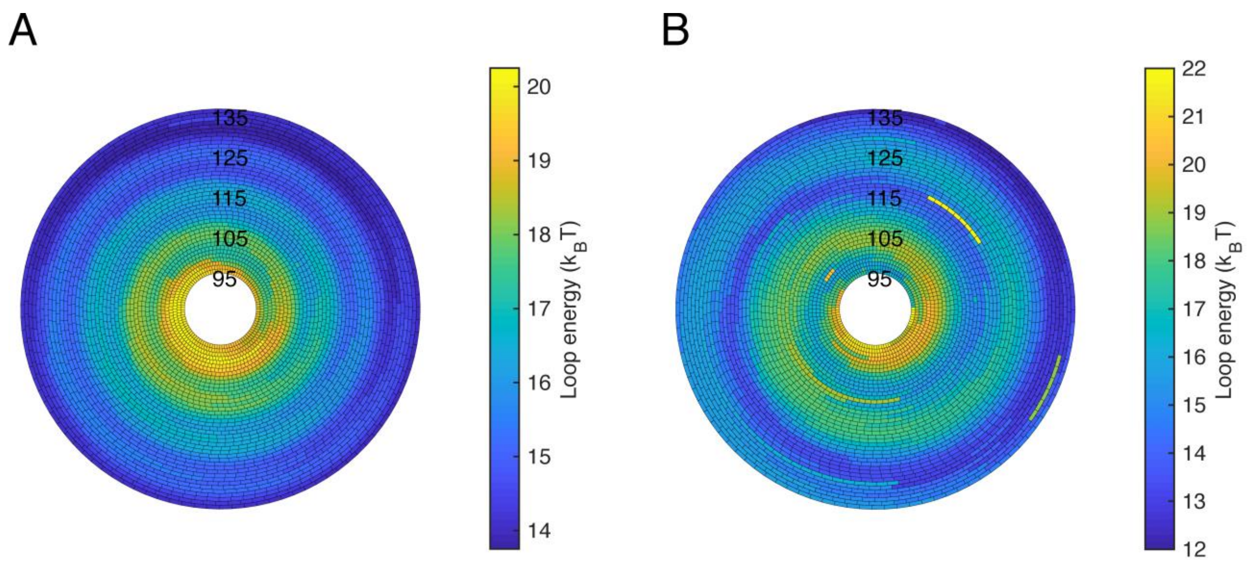

Supplementary Figure S8: Loop energy as a function of bending direction and DNA length (95 bp – 135 bp) with (A) no intrinsic curvature and (B) with the intrinsic curvature of DNA set 1 estimated from the base pair step parameters in [2].

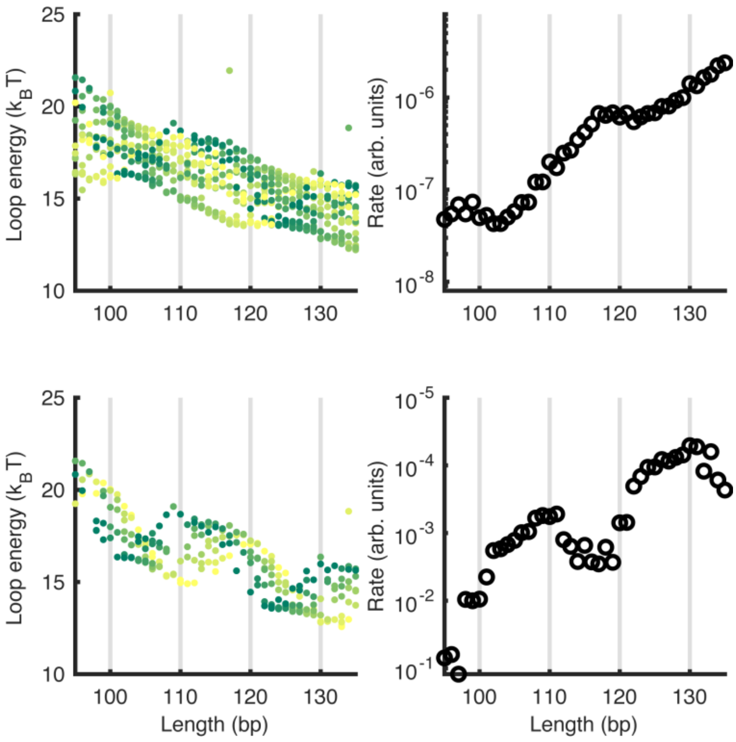

Supplementary Figure S9: *Left column*: The loop energy of DNA set 1 (Supplementary Figure S8 (B)) as a function of length. Different colors (green to yellow) represent different rotational register angles. The top plot shows the full range of register angles (from 0 to 360 degrees) whereas the bottom plot shows a half range (i.e. from 0 to 180 degrees). Restricting the range of register angles is intended to mimic the effect of surface confinement. Each color series shows an oscillation with the same ~20-bp period but a different phase. *Right column*: The looping rate is estimated from the loop energy by carrying out a Boltzmann weighted sum over the full range of register angles (top) or a half range (bottom). These plots show ~20-bp periodicity and a phase shift.

DNA set 1 (5' to 3')	
94 bp	GTGCCAGCAACAGATAGCCACCGGAGCCACACCGGTGCAAACCTCAGCAAGCAGGGTGTGGAAGTAGGACATTTCCCATTCGAGCTCGTTGTAG
96 bp	GTGCCAGCAACAGATAGCCATCCGGAGCCACACCGGTGCAAACCTCAGCAAGCAGGGTGTGGAAGTAGGACATTTTCCCATTCGAGCTCGTTGTAG
98 bp	GTGCCAGCAACAGATAGCCATTCCGGAGCCACACCGGTGCAAACCTCAGCAAGCAGGGTGTGGAAGTAGGACATTTTCCCCATTCGAGCTCGTTGTAG
100 bp	GTGCCAGCAACAGATAGCCACTTCCGGAGCCACACCGGTGCAAACCTCAGCAAGCAGGGTGTGGAAGTAGGACATTTTCACCCATTCGAGCTCGTTGTAG
102 bp	GTGCCAGCAACAGATAGCCAACTTCCGGAGCCACACCGGTGCAAACCTCAGCAAGCAGGGTGTGGAAGTAGGACATTTTCATCCCATTCGAGCTCGTTGTAG
104 bp	GTGCCAGCAACAGATAGCCAAACTTCCGGAGCCACACCGGTGCAAACCTCAGCAAGCAGGGTGTGGAAGTAGGACATTTTCATGCCCATTCGAGCTCGTTGTAG
106 bp	GTGCCAGCAACAGATAGCCATAACTTCCGGAGCCACACCGGTGCAAACCTCAGCAAGCAGGGTGTGGAAGTAGGACATTTTCATGTCCCATTCGAGCTCGTTGTAG
108 bp	GTGCCAGCAACAGATAGCCATTAACTTCCGGAGCCACACCGGTGCAAACCTCAGCAAGCAGGGTGTGGAAGTAGGACATTTTCATGTCCCCATTCGAGCTCGTTGTAG
110 bp	GTGCCAGCAACAGATAGCCAGTTAACTTCCGGAGCCACACCGGTGCAAACCTCAGCAAGCAGGGTGTGGAAGTAGGACATTTTCATGTCACCCATTCGAGCTCGTTGTAG
112 bp	GTGCCAGCAACAGATAGCCACGTTAACTTCCGGAGCCACACCGGTGCAAACCTCAGCAAGCAGGGTGTGGAAGTAGGACATTTTCATGTCAGCCCATTCGAGCTCGTTGTAG
114 bp	GTGCCAGCAACAGATAGCCAGCGTTAACTTCCGGAGCCACACCGGTGCAAACCTCAGCAAGCAGGGTGTGGAAGTAGGACATTTTCATGTCAGGCCCATTCGAGCTCGTTGTAG
116 bp	GTGCCAGCAACAGATAGCCAAGCGTTAACTTCCGGAGCCACACCGGTGCAAACCTCAGCAAGCAGGGTGTGGAAGTAGGACATTTTCATGTCAGGCCCCATTCGAGCTCGTTGTAG
118 bp	GTGCCAGCAACAGATAGCCATAGCGTTAACTTCCGGAGCCACACCGGTGCAAACCTCAGCAAGCAGGGTGTGGAAGTAGGACATTTTCATGTCAGGCCCCCATTCGAGCTCGTTGTAG
120 bp	GTGCCAGCAACAGATAGCCATTAGCGTTAACTTCCGGAGCCACACCGGTGCAAACCTCAGCAAGCAGGGTGTGGAAGTAGGACATTTTCATGTCAGGCCACCCATTCGAGCTCGTTGTAG
122 bp	GTGCCAGCAACAGATAGCCATTTAGCGTTAACTTCCGGAGCCACACCGGTGCAAACCTCAGCAAGCAGGGTGTGGAAGTAGGACATTTTCATGTCAGGCCACCCCATTCGAGCTCGTTGTAG
124 bp	GTGCCAGCAACAGATAGCCACTTTAGCGTTAACTTCCGGAGCCACACCGGTGCAAACCTCAGCAAGCAGGGTGTGGAAGTAGGACATTTTCATGTCAGGCCACTCCCATTCGAGCTCGTTGTAG
126 bp	GTGCCAGCAACAGATAGCCAGCTTTAGCGTTAACTTCCGGAGCCACACCGGTGCAAACCTCAGCAAGCAGGGTGTGGAAGTAGGACATTTTCATGTCAGGCCACTTCCCATTCGAGCTCGTTGTAG
128 bp	GTGCCAGCAACAGATAGCCATGCTTTAGCGTTAACTTCCGGAGCCACACCGGTGCAAACCTCAGCAAGCAGGGTGTGGAAGTAGGACATTTTCATGTCAGGCCACTTCCCCATTCGAGCTCGTTGTAG
130 bp	GTGCCAGCAACAGATAGCCAGTGCTTTAGCGTTAACTTCCGGAGCCACACCGGTGCAAACCTCAGCAAGCAGGGTGTGGAAGTAGGACATTTTCATGTCAGGCCACTTCTCCCATTCGAGCTCGTTGTAG
132 bp	GTGCCAGCAACAGATAGCCAAGTGCTTTAGCGTTAACTTCCGGAGCCACACCGGTGCAAACCTCAGCAAGCAGGGTGTGGAAGTAGGACATTTTCATGTCAGGCCACTTCTTCCCATTCGAGCTCGTTGTAG
134 bp	GTGCCAGCAACAGATAGCCACAGTGCTTTAGCGTTAACTTCCGGAGCCACACCGGTGCAAACCTCAGCAAGCAGGGTGTGGAAGTAGGACATTTTCATGTCAGGCCACTTCTTTCCCATTCGAGCTCGTTGTAG
DNA set 2 (5' to 3')	
94 bp	GTGCCAGCAACAGATAGCCACATGGCAACGAGGTCGCACACGCCCCACACCCAGACCTCCCTGCGAGCGGGCATCCCATTCGAGCTCGTTGTAG
96 bp	GTGCCAGCAACAGATAGCCACCATGGCAACGAGGTCGCACACGCCCCACACCCAGACCTCCCTGCGAGCGGGCATGCCCATTCGAGCTCGTTGTAG
98 bp	GTGCCAGCAACAGATAGCCAGCCATGGCAACGAGGTCGCACACGCCCCACACCCAGACCTCCCTGCGAGCGGGCATGGCCCATTCGAGCTCGTTGTAG
100 bp	GTGCCAGCAACAGATAGCCACGCCATGGCAACGAGGTCGCACACGCCCCACACCCAGACCTCCCTGCGAGCGGGCATGGGCCCATTCGAGCTCGTTGTAG
102 bp	GTGCCAGCAACAGATAGCCATCGCCATGGCAACGAGGTCGCACACGCCCCACACCCAGACCTCCCTGCGAGCGGGCATGGGTCCCATTCGAGCTCGTTGTAG
104 bp	GTGCCAGCAACAGATAGCCAATCGCCATGGCAACGAGGTCGCACACGCCCCACACCCAGACCTCCCTGCGAGCGGGCATGGGTACCCATTCGAGCTCGTTGTAG
106 bp	GTGCCAGCAACAGATAGCCAGATCGCCATGGCAACGAGGTCGCACACGCCCCACACCCAGACCTCCCTGCGAGCGGGCATGGGTACCCCATTCGAGCTCGTTGTAG
108 bp	GTGCCAGCAACAGATAGCCACGATCGCCATGGCAACGAGGTCGCACACGCCCCACACCCAGACCTCCCTGCGAGCGGGCATGGGTACACCCATTCGAGCTCGTTGTAG
110 bp	GTGCCAGCAACAGATAGCCAGCGATCGCCATGGCAACGAGGTCGCACACGCCCCACACCCAGACCTCCCTGCGAGCGGGCATGGGTACAACCCATTCGAGCTCGTTGTAG

112 bp	GTGCCAGCAACAGATAGCCACGCGATCGCCATGGCAACGAGGTCGCACACGCCCCACACCCAGACCTCCCTGCGAGCGGGCATGGGTACAATCCCATTCGAGCTCGTTGTAG
114 bp	GTGCCAGCAACAGATAGCCAGCGCGATCGCCATGGCAACGAGGTCGCACACGCCCCACACCCAGACCTCCCTGCGAGCGGGCATGGGTACAATGCCCATTCGAGCTCGTTGTAG
116 bp	GTGCCAGCAACAGATAGCCACGCGCGATCGCCATGGCAACGAGGTCGCACACGCCCCACACCCAGACCTCCCTGCGAGCGGGCATGGGTACAATGTCCCATTCGAGCTCGTTGTAG
118 bp	GTGCCAGCAACAGATAGCCAACGCGCGATCGCCATGGCAACGAGGTCGCACACGCCCCACACCCAGACCTCCCTGCGAGCGGGCATGGGTACAATGTCCCCATTCGAGCTCGTTGTAG
120 bp	GTGCCAGCAACAGATAGCCACACGCGCGATCGCCATGGCAACGAGGTCGCACACGCCCCACACCCAGACCTCCCTGCGAGCGGGCATGGGTACAATGTCCCCCATTCGAGCTCGTTGTAG
122 bp	GTGCCAGCAACAGATAGCCACCACGCGCGATCGCCATGGCAACGAGGTCGCACACGCCCCACACCCAGACCTCCCTGCGAGCGGGCATGGGTACAATGTCCCCCCATTCGAGCTCGTTGTAG
124 bp	GTGCCAGCAACAGATAGCCACCCACGCGCGATCGCCATGGCAACGAGGTCGCACACGCCCCACACCCAGACCTCCCTGCGAGCGGGCATGGGTACAATGTCCCCCCCATTCGAGCTCGTTGTAG
126 bp	GTGCCAGCAACAGATAGCCAACCCACGCGCGATCGCCATGGCAACGAGGTCGCACACGCCCCACACCCAGACCTCCCTGCGAGCGGGCATGGGTACAATGTCCCCGCCCATTCGAGCTCGTTGTAG
128 bp	GTGCCAGCAACAGATAGCCACACCCACGCGCGATCGCCATGGCAACGAGGTCGCACACGCCCCACACCCAGACCTCCCTGCGAGCGGGCATGGGTACAATGTCCCCGTCCCATTCGAGCTCGTTGTAG
130 bp	GTGCCAGCAACAGATAGCCAACACCCACGCGCGATCGCCATGGCAACGAGGTCGCACACGCCCCACACCCAGACCTCCCTGCGAGCGGGCATGGGTACAATGTCCCCGTTCCCATTCGAGCTCGTTGTAG
132 bp	GTGCCAGCAACAGATAGCCACACACCCACGCGCGATCGCCATGGCAACGAGGTCGCACACGCCCCACACCCAGACCTCCCTGCGAGCGGGCATGGGTACAATGTCCCCGTTGCCCATTCGAGCTCGTTGTAG
134 bp	GTGCCAGCAACAGATAGCCACCACACCCACGCGCGATCGCCATGGCAACGAGGTCGCACACGCCCCACACCCAGACCTCCCTGCGAGCGGGCATGGGTACAATGTCCCCGTTGCCCCATTCGAGCTCGTTGTAG
Full sticky ends PCR primer pairs (5' to 3')	
Forward	TGAATTTACG[Cy5dT]GCCAGCAACAGA[BiotindT]AGC
Reverse	GTAAATTCAC[Cy3dT]ACAACGAGCTCGAATGGG
Gapped sticky ends PCR primer pairs (5' to 3')	
Forward	TGAATTTACGCTG[Cy5dT]GCCAGCAACAGA[BiotindT]AGCCA
Reverse	[Cy3]GTAAATTCACGACTACAACGAGCTCGAATGGG
PCR primer for the terminal immobilization (5' to 3')	
Forward	[BiotinTEG]GAAACAT[iCy5]ATGAATTTACCGTGCCAGCAACAGATAGC
Blocking oligos for making partial duplexes (5' to 3')	
Blocking-Cy5full	GCTATCTGTTGCTGGCAC
Blocking-Cy5gap	TGGCTATCTGTTGCTGGCAC
Blocking-Cy3	CCCATTCGAGCTCGTTGTAG

Supplementary Table S1: List of DNA sequences, PCR primers, and blocking oligonucleotides. All molecules in DNA sets 1 and 2 include the common adapter sequences (20 bp) at both ends, which are also present in the PCR primer pairs. PCR primers and blocking oligos are hybridized to each other to make the partial duplexes that are sticky on one end and blunt on the other: Blocking-Cy5full and Blocking-Cy5gap are hybridized with the forward primers of full and gapped sticky ends, and Blocking-Cy3 hybridizes with the reverse primers of both full and gapped sticky ends, respectively. These partial duplexes are used to measure the association rate (k_{on}) between the sticky ends and the lifetime (τ_{on}) of the linker duplex.

Supplementary References

1. Le, T. T. and Kim, H. D. (2013). Measuring Shape-Dependent Looping Probability of DNA. *Biophysical Journal*, 104, 2068–2076.
2. Zuiddam, M., Everaers, R., & Schiessel, H. (2017). Physics behind the mechanical nucleosome positioning code. *Physical Review E*, 96(5), 052412.
